# Low Absolute Lymphocyte Counts in the Peripheral Blood Predict Inferior Survival and Improve the International Prognostic Index in Testicular Diffuse Large B-Cell Lymphoma

**DOI:** 10.3390/cancers12071967

**Published:** 2020-07-20

**Authors:** Pauli Vähämurto, Marjukka Pollari, Michael R. Clausen, Francesco d’Amore, Sirpa Leppä, Susanna Mannisto

**Affiliations:** 1 Department of Oncology, Helsinki University Hospital, Comprehensive Cancer Center, 00290 Helsinki, Finland; pauli.vahamurto@helsinki.fi (P.V.); sirpa.leppa@helsinki.fi (S.L.); 2 Applied Tumor Genomics Research Program, Faculty of Medicine, University of Helsinki, 00014 Helsinki, Finland; Marjukka.Pollari@pshp.fi; 3 Department of Oncology, Tampere University Hospital, 33520 Tampere, Finland; 4 Department of Hematology, Vejle Hospital, 7100 Vejle, Denmark; Michael.Roost.Clausen@rsyd.dk; 5 Department of Hematology, Aarhus University Hospital, 8200 Aarhus, Denmark; frandamo@rm.dk

**Keywords:** diffuse large B-cell lymphoma, testicular lymphoma, extranodal lymphoma, absolute lymphocyte count, lymphopenia, CNS prophylaxis

## Abstract

Low absolute lymphocyte counts (ALC) and high absolute monocyte counts (AMC) are associated with poor survival in patients with diffuse large B-cell lymphoma (DLBCL). We studied the prognostic impact of the ALC and AMC in patients with testicular DLBCL (T-DLBCL). T-DLBCL patients were searched using Southern Finland University Hospital databases and the Danish lymphoma registry. The progression free survival (PFS) and overall survival (OS) were assessed using Kaplan-Meier and Cox proportional hazards methods. We identified 178 T-DLBCL patients, of whom 78 (44%) had a low ALC at diagnosis. The ALC did not correlate with survival in the whole cohort. However, among the patients treated with rituximab (R) containing regimen, a pre-therapeutic low ALC was associated with an increased risk of progression (HR 1.976, 95% CI 1.267–3.086, *p* = 0.003). Conversely, intravenous (iv) CNS directed chemotherapy translated to favorable outcome. In multivariate analyses, the advantage of an iv CNS directed chemotherapy was sustained (PFS, HR 0.364, 95% CI 0.175–0.757, *p* = 0.007). The benefit of R and intravenous CNS directed chemotherapy was observed only in non-lymphopenic patients. The AMC did not correlate with survival. A low ALC is an adverse prognostic factor in patients with T-DLBCL. Alternative treatment options for lymphopenic patients are needed.

## 1. Introduction

Testicular diffuse large B-cell lymphoma (T-DLBCL) is a rare lymphoid cancer found in elderly men, and the most common testicular malignancy in men over 60 years. T-DLBCL has a tendency to spread to other extranodal sites, including the contralateral testis and central nervous system (CNS). The addition of CD20 antibody rituximab (R) to standard cyclophosphamide, doxorubicin, vincristine and prednisone (CHOP) chemotherapy has not resulted in as significant improvement in survival among the patients with T-DLBCL as it has in the patients with nodal diffuse large B-cell lymphoma (DLBCL) [1,2,3,4,5]. However, more aggressive CNS directed chemotherapy with high dose methotrexate (MTX) and/or cytarabine (AraC) has been associated with a better survival and lower risk of CNS recurrence in patients with high risk DLBCL, and also in T-DLBCL [5,6,7,8]. In our T-DLBCL cohort, CNS directed chemotherapy correlated with an improved survival. However, the effect may be due to the improved control of the systemic disease rather than the reduction in the low number of CNS relapses. Additionally, our results indicated an improved survival in response to either the surgical removal or irradiation of the contralateral testis protected that, as protected by blood–testis barrier, might serve as a sanctuary for the malignant cells [9].

As in DLBCL, a high stage, advanced age at diagnosis, high serum lactate dehydrogenase (LDH) and involvement of extranodal sites other than testis have an adverse prognostic impact on survival in patients with T-DLBCL. Together with the Eastern Cooperative Oncology Group (ECOG) performance score, these factors form the International Prognostic Index (IPI), which is the most used clinical risk stratification model in lymphoma, including T-DLBCL [7,10,11]. However, for particular DLBCL subsets, e.g., primary central nervous system lymphoma (PCNSL), with extranodal involvements associated with specific clinical and therapeutic implications, adapted models have been proposed to improve subset-specific prognostication [12,13,14].

With the emerging biological data, aggressive B-cell lymphomas are further divided into molecular entities [15] with different clinical behaviors. According to gene expression profiling, the majority of T-DLBCLs resemble the activated B-cell-like (ABC) subtype of nodal DLBCLs, and based on mutational landscape and immunophenotypic profiles, immune-escape and sustained NF-κ-B signaling emerge as prominent biological features [16].

Additionally, the host immune response is emerging as a prognostic factor in nodal DLBCL [17]. In particular, tumor infiltrating macrophages and T lymphocytes have a prognostic impact on survival in patients treated with immunochemotherapy [17,18,19,20,21]. Emerging evidence also indicates that circulating host immunity is associated with the outcome. In particular, a low absolute lymphocyte count (ALC) at diagnosis has an adverse prognostic impact on survival in DLBCL patients, independent from the molecular subtype [22]. Lymphopenic DLBCL patients appear not to benefit as much from the addition of R to chemotherapy as non-lymphopenic patients [23], although the introduction of R was reported to lead to improvements in DLBCL patients with a low ALC (<1.0 × 10^9^/L) as compared to those with a high ALC [24]. In fact, in DLBCL patients, relative lymphopenia has been associated w a poorer survival not only when found as a pre-treatment parameter, but also when identified during treatment, after first line therapy or at the time of relapse [22,25,26].

A high absolute monocyte count (AMC) has also been linked to a shorter survival in patients with DLBCL, also in R-era [27,28]. In particular, the prognostic significance of AMCs has been shown when applying the lymphocyte to monocyte ratio (LMR) [27,29,30,31]. A low LMR has been shown to have an adverse prognostic impact on survival both in R-CHOP and CHOP treated patients [29,30,31,32]. Additionally, the LDH to ALC ratio has been reported to predict outcomes in DLBCL [33].

We have recently shown that a high number of tumor infiltrating macrophages and lymphocytes are associated with an improved survival in patients with T-DLBCL [34,35]. The aim of this study was to investigate the prognostic impact of circulating host immune cells, including the ALC and AMC in T-DLBCL, in response to rituximab and CNS directed chemotherapy and to assess their ability to improve on the standard IPI in T-DLBCL.

## 2. Results

### 2.1. Patient Demographics and Survival According to the ALC

Altogether, 178 patients treated with CHOP or CHOP-like chemotherapy and ALC data available were identified. The median age at diagnosis was 69 years (range 37–88). The median follow up time was 60 months. The median progression free survival (PFS) was 47 months (range 0.27–60) and the median overall survival (OS) was 55 months (range 0.27–60). The median ALC at diagnosis was 1.385 × 10^9^/L (range 0.196 × 10^9^/L–10.600 × 10^9^/L). The patient characteristics are shown in Table 1. R containing immunochemotherapy was given to 109 patients, CNS directed therapy to 113 patients, and CNS directed therapy in combination with immunochemotherapy to 78 patients. The timing and the dose of the CNS directed chemotherapy varied according to the protocol used, with a minimum cumulative dose of intravenous (iv) MTX 1.5 g/m^2^ and cytarabine 8 g/m^2^ for two cycles. In addition, the contralateral testis was prophylactically treated either by irradiation of orchiectomy in 85 (58%) patients. The patient characteristics of the immunochemotherapy treated subcohort were similar to those of the entire cohort (Table 1). In this subcohort, the IPI and its factors apart from age (PFS) and a high LDH (OS) correlated with a shorter survival (Table 2).

**Figure 1:**
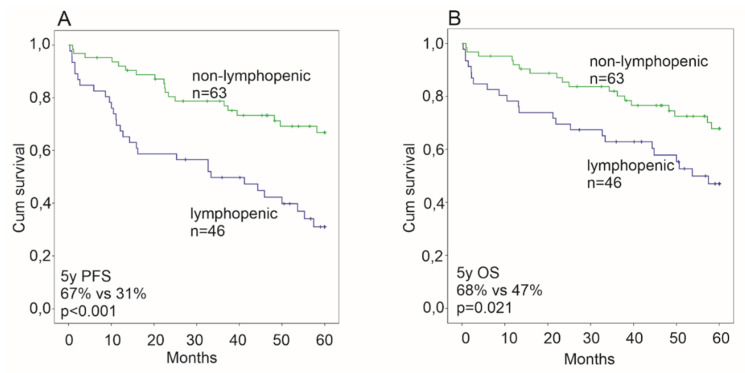
Kaplan-Meier survival estimates of the impact of lymphopenia on survival among rituximab treated patients for progression free survival (PFS, **A**) and overall survival (OS, **B**). Lymphopenia predicts an inferior survival for both OS and PFS.

Lymphopenia (ALC < 1.3 × 10^9^/L) was associated with an advanced stage, elevated LDH and a high IPI (IPI > 2), but not with the T-cell composition of the microenvironment (Table 1). The R and CNS directed chemotherapy and treatment of the contralateral testis were equally distributed between the ALC low and high subgroups (Table 1). The ALC did not correlate with survival when analyzed in the entire cohort taken as one group. However, in the 109 T-DLBCL patients treated with immunochemotherapy, lymphopenic patients had an inferior outcome (5-year PFS 31%, 5-year OS 47%) compared to non-lymphopenic patients (5-year PFS 67%, 5-year OS 68%, *p* < 0.001, *p* = 0.021, respectively; Figure 1). In Cox univariate analyses, the risk of progression and death were 2.9- and 2.0-fold for lymphopenic patients (Table 2). A multivariate analysis confirmed the ALC as an independent prognostic factor for progression (Table 3).

**Figure 2:**
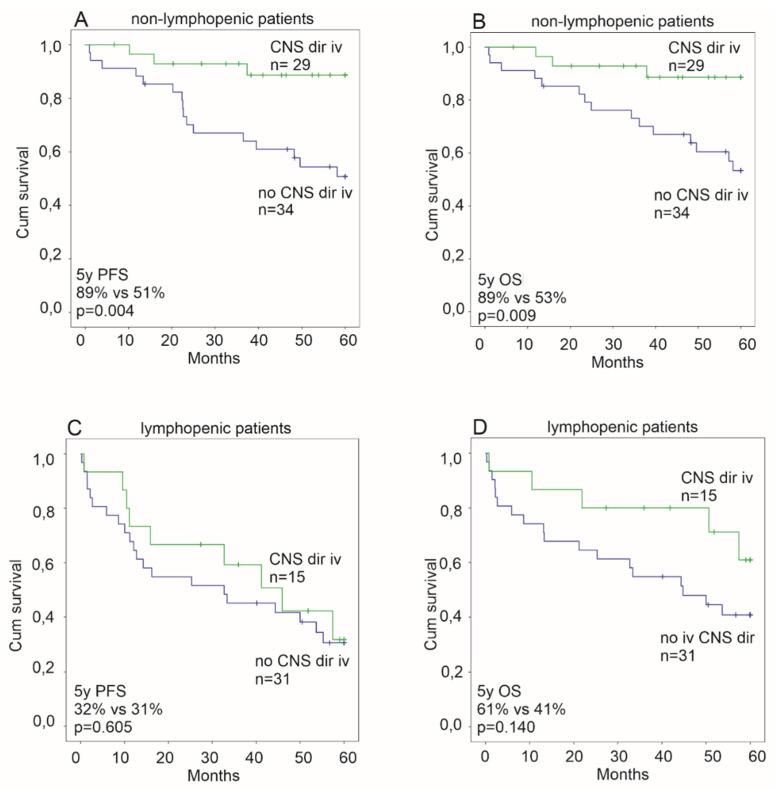
Kaplan-Meier estimates of the impact of intravenous (iv) central nervous system directed chemotherapy on survival among non-lymphopenic rituximab treated patients (**A**,**B**) and lymphopenic rituximab treated patients (**C**,**D**). An association to an improved progression free survival (PFS) and overall survival (OS) is seen only among non-lymphopenic patients (**A**,**B**).

Next we studied the impact of CNS directed treatment on the outcome among the patients treated with immunochemotherapy. In Cox regression analyses (Table 2 and Table 3), iv CNS directed treatment associated independently with a longer survival, as previously reported [9]. However, when interaction with the ALC was tested, iv CNS directed chemotherapy was associated with an improved PFS and OS in the non-lymphopenic patients (iv CNS dir treated; PFS, HR 0.198 95% CI 0.058–0.678, *p* = 0.010; OS, HR 0.223 95% CI 0.064–0.769, *p* = 0.018), whereas no benefit was seen in the lymphopenic patients (iv CNS dir treated; PFS, HR 0.814 95% CI 0.373–1.779, *p* = 0.606; OS HR 0.482, 95% CI 0.179–1.300, *p* = 0.149 (Figure 2)).

As previously reported [9], intrathecal (it) CNS chemotherapy did not have any impact on survival among the immunochemotherapy cohort. In this study, the findings were sustained despite the ALC status.

**Figure 3:**
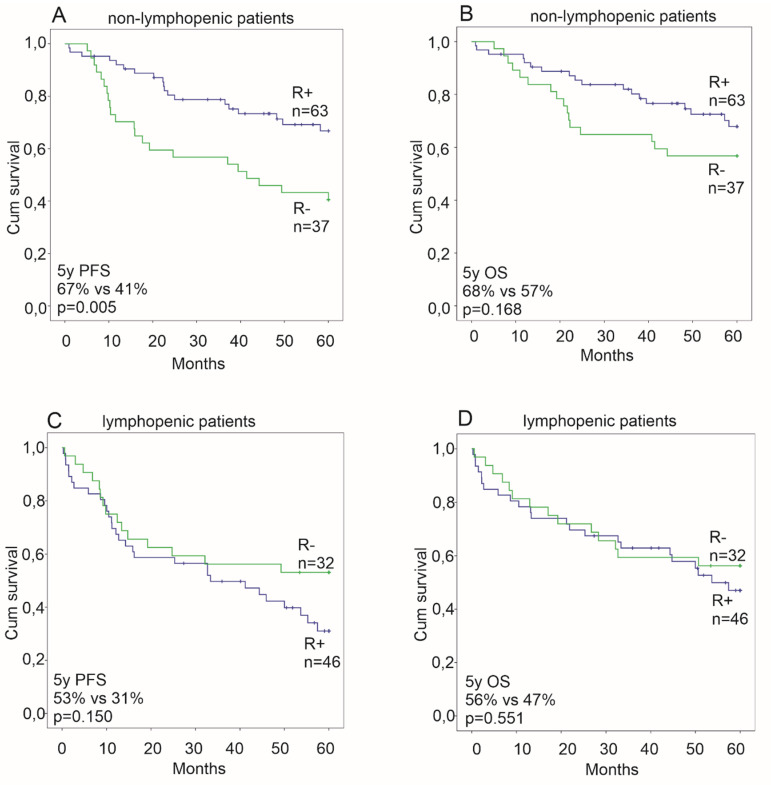
Kaplan-Meier survival estimates comparing patients treated with rituximab (R+) and patients treated before rituximab era (R-era) among non-lymphopenic patients (**A**,**B**) and lymphopenic patients (**C**,**D**). Association with an improved progression free survival (PFS) is seen among non-lymphopenic patients (**A**) without association to the improved overall survival (OS). Among lymphopenic patients no differences in survival were seen.

We also compared survival according to the ALC between the patients treated prior to or after the introduction of R in the standard treatment of T-DLBCL. Similarly to iv CNS directed chemotherapy, the benefit of adding R to chemotherapy was only observed in non-lymphopenic patients (Figure 3).

However, the prophylactic treatment of the contralateral testis either by orchiectomy or irradiation translated to a better survival both in lymphopenic and non-lymphopenic patients (Table 2 and Table 3, Figure S1).

### 2.2. Patient Demographics and Survival According to the AMC and LMR

The AMC was available for 156 T-DLBCL patients treated with CHOP or CHOP-like treatment with or without R. The median age at diagnoses for these patients was 70 years (range 37–88). The median AMC was 0.520 × 10^9^/L (range 0.100 × 10^9^/L–1.680 × 10^9^/L). The median PFS for these patients was 44 months (range 0.57–60) and the median OS was 54 months (range 0.57–60). For the patients treated with R containing immunochemotherapy, and with the AMCs available (*n* = 95), the median age at diagnosis was 70 years (range 37–88). The median AMC in this patient group was 0.530 × 10^9^/L (range 0.100 × 10^9^/L–1.680 × 10^9^/L), the median PFS was 45 months (range 0.77–60) and the median OS was 52 months (range 0.77–60).

The AMC was not associated with survival in the entire cohort nor among the subgroup of patients treated with immunochemotherapy.

We also looked at the ratio of the ALC to AMC (lymphocyte to monocyte ratio, LMR). While the LMR as a continuous variable did not correlate with the outcome, an association between LMR < 3:1 and survival was observed among the patients treated with immunochemotherapy (PFS, HR 3.500, 95% CI 1.630–7.515, *p* = 0.001; OS HR 3.406, 95% CI 1.463–7.928, *p* = 0.004 (Table S1)). Furthermore, according to Kaplan-Meier estimates, the patients with a LMR < 3:1 had an inferior outcome (5-year PFS 38%, 5-year OS 52%) compared to ones with a high LMR (5-year PFS 72%, 5-year OS 74%, *p* = 0.003, *p* = 0.033, respectively; Figure S2).

### 2.3. Survival According to the LDH to ALC Ratio

In our cohort of T-DLBCL patients, the LDH to ALC ratio was recognized as a potential prognostic factor in univariate analysis (Table 2), but in multivariate analysis with other IPI factors it did not hold its prognostic value for either PFS or OS (Table S2).

## 3. Discussion

Emerging evidence indicates that the circulating host immunity is an independent prognostic factor in nodal DLBCL, most likely reflecting the immune status of the host and tumor microenvironment. In this respect, previous studies have demonstrated that the circulating immune cells measured by the complete blood counts, such as the ALC and AML, predict the outcome of patients with nodal DLBCL [22,25,28,29,30,31]. In fact, a peripheral lymphopenia has been consistently reported to impact on patients’ PFS and OS in a variety of hematopoietic and non-hematopoietic malignancies [36].

In this retrospective study, we report lymphopenia as an independent adverse prognostic factor for patients with T-DLBCL. In our previous study, we showed that a T-cell inflamed tumor microenvironment predicts a favorable outcome in Finnish T-DLBCL patients also included in this study [35]. However, we did not find a correlation between the peripheral blood ALC and the amount of specific tumor infiltrating T-cells, and could therefore not propose that the blood ALC serves as a surrogate marker for the composition of the tumor microenvironment. However, this suggests other roles of the host immunity in treatment responses other than the amount and quality of the immune effector cells in the immediate vicinity of the malignant cells.

We did not identify the AMC as a prognostic factor in T-DLBCL, whereas the LMR was identified as an independent prognostic factor. As the LMR was largely a derivative of the ALC in our cohort, it did not provide independent prognostic information on our cohort.

Lymphopenia in our series was associated with known clinical risk factors linked to a high tumor burden, such as a high LDH and IPI (Table 1). It remains to be shown, whether a high tumor burden is a result from the impaired host immunity incapable of controlling the tumor cell proliferation or lymphopenia a result from the high tumor burden exhausting the immune system, or an interplay of these factors leading to a high risk lymphoma with a poor treatment response.

However, in multivariate analyses with other risk factors and chemotherapy intensity among R treated patients, the low ALC retained its independent adverse prognostic value (Table 3). This implies that a low ALC also has a negative impact on treatment efficacy. Additionally, the high LDH to ALC ratio, that has been reported to predict the outcome in DLBCL [33], associated with the prognosis in a univariate analysis among R treated patients in our cohort. The prognostic impact, however, was lost in a multivariate analysis with other IPI factors and chemotherapy intensities.

In our cohort, the PFS was better in the non-lymphopenic patients treated with a R containing regimen when compared to the patients treated with chemotherapy only, whereas lymphopenic T-DLBCL patients appeared not to benefit from the addition of R to chemotherapy. Similar results have been observed in studies on nodal DLBCL [23]. Indeed, lymphocytes, and especially T-cells and NK-cells, have been hypothesized to have a role in the mechanism of action of R [22,32,35,37,38,39], which could explain why the survival of lymphopenic patients has not improved in the immunochemotherapy era. Since the administration of R in our series was a consequence of the systematic change in therapy, the patient demographics other than lymphopenia are not considered to have an effect on this improvement in outcome.

As the patients with T-DLBCL have a high risk of CNS progression, 39% of the patients in our cohort received iv CNS directed chemotherapy as part of their treatment. Previously we have shown that this type of CNS prophylaxis is associated with improved survival [9]. In this further analysis, we observed that the survival benefit in response to a more aggressive chemotherapy was only seen in non-lymphopenic patients. Specifically, in the non-lymphopenic patients, the subgroup receiving immunochemotherapy with iv CNS prophylaxis had a longer PFS and OS, whereas this benefit was not seen in lymphopenic patients (Figure 2). The retrospective analysis includes the risk for selection bias, as younger and more fit patients tend to be treated more aggressively. However, lymphopenia in our cohort was not associated with a high age, poor performance status, or different treatment modalities, and the performance status was not associated with the treatment selection in this cohort [9].

The independent additional prognostic information provided by the ALC parameter improved the outcome prediction power of standard IPI in T-DLBCL patients. Our results highlight the unmet clinical need for T-DLBCL, in particular for the lymphopenic patients. They had inferior survival rate even when treated with more aggressive immunochemotherapy containing iv CNS penetrating chemotherapy, and new treatment options are required.

Chapuy et al. have reported the overexpression of programmed cell death protein 1 (PD-1) ligands in seven T-DLBCL patients [40]. Interestingly, PD-1 inhibition has shown efficacy in a small series of patients with relapsed/refractory primary CNS lymphoma and T-DLBCL patients with CNS relapse [41]. However, as we reported previously, only 34% of T-DLBCL cells express PD-L1 [34], and also a low ALC in peripheral blood has been associated with an inferior response to PD-1 inhibition [36]. Ongoing clinical trials, e.g., with check point inhibitors, lenalidomide and chimeric antigen receptor (CAR) T-cell therapy, will show how the patients with a less favorable host immunity and tumor microenvironment profile will benefit from treatments aiming at activation of the immune system.

## 4. Materials and Methods

### 4.1. Patients

The pathology databases of three Southern Finland University Hospitals and the Danish Lymphoma Registry (LYFO) were searched for testicular DLBCL patients. Only patients with a testicular involvement of DLBCL at first diagnosis were included in the analysis and patients with primary CNS lymphoma were excluded. Data on patient demographics and clinic-pathologic characteristics were recorded. The ALC and AMC were obtained from routine pre-therapeutic peripheral blood counts. CNS directed systemic therapy was defined as a minimum iv MTX of 1.5 g/m^2^ given in two courses or an iv AraC of 8 g/m^2^ (given 2–3 g/m^2^ four times every 12 h) in two courses, or a combination of MTX and AraC.

For the statistical analysis of the ALCs, the cohort was divided into a “low ALC” and a “high ALC” subset using the lower limit for the normal lymphocyte count (ALC = 1.3 × 10^9^/L) from the reference laboratory. For the analyses of the LMR, the earlier reported cut-off ratio of 3.0:1.0 was used [32] and the LDH to ALC had an earlier reported cut-off ratio of 400 [33].

### 4.2. Survival Definitions and Statistical Analysis

SPSS Statistics 22 (IBM Corp. Released 2013. IBM SPSS Statistics for Windows, Version 22.0. Armonk, NY, USA.) was used for statistical analyses. The χ^2^ test was used to assess the differences in the frequency of the baseline characteristics and treatment modalities. Univariate and multivariate analyses were performed according to the Cox proportional hazards regression model. Survival rates were estimated using the Kaplan-Meier method, and the differences were compared with log-rank test. The probability values below 0.05 were considered statistically significant. All comparisons were two-tailed.

### 4.3. Ethical Considerations

All the patient data were handled according to the Good Scientific Practice (GSP) Guidelines. For the Finnish cohort, the study was approved by the Ethics Committee in Helsinki University Hospital, which waived the requirement to obtain informed consent. The Registration in the Danish Lymphoma Registry (LYFO) is compliant with Danish regulations and approved by the National Board of Health and the Danish Data Protection Agency.

## 5. Conclusions

We introduce the ALC as an easily accessible prognostic marker for the patients with testicular DLBCL in the R-era. Our data on T-DLBCL show that lymphopenia is an independent prognostic marker. Non-lymphopenic patients benefit from R and CNS directed treatment, whereas a clear benefit of these was not recorded in lymphopenic patients.

## Figures and Tables

**Table 1:** Patient baseline characteristics.

Characteristic	All Patients				Patients Treated with Immunochemotherapy			
	All patients, *n* = 178 (100%)	Patients with ALC < 1.3 × 10^9^/L, *n* = 78 (100%)	Patients with ALC ≥ 1.3 × 10^9^/L, *n* = 100 (100%)	*p*	All patients, *n* = 109 (100%)	Patients with ALC < 1.3 × 10^9^/L, *n* = 46 (100%)	Patients with ALC ≥ 1.3 × 10^9^/L, *n* = 63 (100%)	*p*
Age over 60	133 (75)	60 (77)	73 (73)	0.550	87 (80)	39 (85)	48 (76)	0.270
Stage ≥3	62 (35), missing 1	38 (48), missing 1	25 (25)	**0.001**	42 (38)	25 (54)	17 (27)	**0.004**
ECOG ≥2	23 (13)	14 (18)	9 (9)	0.077	16 (15)	10 (22)	6 (10)	0.075
LDH high	58 (33), missing 2	34 (44)	24 (25)	**0.007**	39 (36)	23 (50)	16 (25)	**0.008**
Number of extranodal sites >1	37 (21), missing 1	20 (26)	17 (17)	0.169	24 (22)	14 (30)	10 (16)	0.070
IPI ≥3	49 (28), missing 1	32 (41)	18 (18)	**<0.001**	34 (31)	22 (48)	12 (19)	**0.002**
T-cell signature analyzed [35]	53 (30)	17 (22)	36 (36)	0.301	27 (25)	8 (17)	19 (30)	0.616
high/interm	42 (79)	12 (71)	30 (83)		22 (81)	2 (25)	16 (84)	
low	11 (21)	5 (29)	6 (17)		5 (19)	6 (75)	3 (16)	
Rituximab as part of treatment	109 (61)	46 (59)	63 (63)	0.584	109 (100)	46 (100)	63 (100)	
CNS directed treatment;	113 (63)	48 (62)	65 (65)	0.784	78 (72)	31 (67)	47 (75)	0.368
iv-therapy	69 (39)	28 (36)	41 (41)		44 (40)	15 (33)	29 (46)	
it-therapy	44 (25)	20 (26)	24 (24)		34 (31)	16 (35)	18 (29)	
none	65 (37)	30 (39)	35 (35)		31 (28)	15 (33)	16 (25)	
Treatment of the contralateral testis	85 (58) (missing 2)	33 (42)	52 (53)	0.226	60 (56) (missing 1)	23 (51)	37 (62)	0.441

ALC, absolute lymphocyte count; ECOG, Eastern Cooperative Oncology Group performance score; LDH, lactate dehydrogenase; CNS, central nervous system; iv, intravenous; it, intrathecal; IPI, International Prognostic Index. Statistically significant *p*-values are in bold.

**Table 2:** Cox regression analyses on the univariate level of rituximab treated patients showing the association of baseline characteristics and treatment parameters with the outcome.

Parameter	PFS, Hazard Ratio (95% CI)	*p*	OS, Hazard Ratio (95% CI)	*p*
low ALC	2.857 (1.605–5.084)	**<0.001**	2.038 (1.100–3.778)	**0.024**
low LMR	2.760 (1.356–2.760)	**0.005**	2.238 (1.044–4.796)	**0.038**
high LDL/ALC	3.574 (1.891–6.756)	**<0.001**	3.075 (1.504–6.287)	**0.002**
Age >60	2.390 (0.947–6.034)	0.065	5.784 (1.395–23.981)	**0.016**
Stage ≥3	2.046 (1.167–3.588)	**0.013**	2.099 (1.136–3.875)	**0.018**
ECOG ≥2	3.328 (1.724–6.424)	**<0.001**	3.614 (1.802–7.249)	**<0.001**
LDH high	1.962 (1.115–3.450)	**0.019**	1.720 (0.927–3.189)	0.085
extranodal sites >1	2.739 (1.500–5.001)	**0.001**	3.224 (1.697–6.124)	**<0.001**
IPI ≥3	2.741 (1.557–4.824)	**<0.001**	0.331 (0.179–0.613)	**<0.001**
iv CNS dir	0.421 (0.219–0.807)	**0.009**	0.318 (0.147–0.688)	**0.004**
Treatment of the contralateral testis	0.423 (0.237–0.756)	**0.004**	0.402 (0.212–0.764)	**0.005**

PFS, progression free survival; OS, overall survival; ALC, absolute lymphocyte count; LMR, lymphocyte-to-monocyte ratio; Age >60 y, patients over 60 years; ECOG, Eastern Cooperative Oncology Group performance score; LDH, lactate dehydrogenase; IPI, International Prognostic Index; iv CNS dir, intravenous central nervous system directed treatment. Statistically significant *p*-values are in bold.

**Table 3:** Cox regression analyses on the multivariate level of rituximab treated patients showing an independent association of baseline characteristics and treatment parameters with the outcome.

Parameter	PFS, Hazard Ratio (95% CI)	*p*	OS, Hazard Ratio (95% CI)	*p*
low ALC	1.960 (1.064–3.610)	**0.031**	1.416 (0.732–2.738)	0.302
Age >60 y	1.646 (0.622–4.355)	0.316	4.244 (0.93–18.136)	0.051
Stage ≥3	0.703 (0.313–1.581)	0.394	0.765 (0.334–1.754)	0.527
ECOG ≥2	1.584 (0.745–3.366)	0.232	1.576 (0.704–3.525)	0.268
LDH high	1.825 (0.897–3.712)	0.097	1.767 (0.819–3.809)	0.147
extranodal sites >1	3.224 (1.483–7.010)	**0.003**	4.149 (1.858–9.266)	**0.001**
iv CNS dir	0.398 (0.191–0.829)	**0.014**	0.283 (0.123–0.651)	**0.003**
Treatment of the contralateral testis	0.455 (0.250–0.830)	**0.010**	0.283 (0.123–0.651)	**0.021**

PFS, progression free survival; OS, overall survival; ALC, absolute lymphocyte count; Age >60 years, patients over 60 years; ECOG, Eastern Cooperative Oncology Group performance score; LDH, lactate dehydrogenase level; IPI, International Prognostic Index; iv CNS dir, intravenous central nervous system directed treatment. Statistically significant *p*-values are bolded.

## Supplementary Materials

The following are available online at https://www.mdpi.com/2072-6694/12/7/1967/s1, Figure S1: Kaplan-Meier estimates of the impact of treatment of the contralateral testis on survival among non-lymphopenic, rituximab treated patients (A, B); lymphopenic rituximab treated patients (C, D); Figure S2: Kaplan-Meier estimates of the impact of lymphocyte to monocyte ratio (LMR) among rituximab treated patients showing improved overall survival (OS) (A) and progression free survival (PFS) (B) among patients with LMR ≥ 3:1, Table S1: Cox regression analyses on multivariate level of rituximab treated patients showing independent association of lymphocyte-monocyte ratio and other baseline characteristics and treatment parameters with outcome; Table S2: Cox regression analyses on multivariate level of rituximab treated patients showing independent association of high lactate dehydrogenase to lymphocyte ratio and other baseline characteristics and treatment parameters with outcome.
