# Supplementary material for: Low Absolute Lymphocyte Counts in the Peripheral Blood Predict Inferior Survival and Improve the International Prognostic Index in Testicular Diffuse Large B-Cell Lymphoma
Source: Cancers (Basel). 2020 Jul 20;12(7):1967. doi: 10.3390/cancers12071967 (PMC7409117; doi:10.3390/cancers12071967)
Supplement: Supplementary file 1 [file cancers-12-01967-s001.pdf]

cancers

Supplementary Materials

Low Absolute Lymphocyte Counts in the Peripheral Blood Predict Inferior Survival and Improve the International Prognostic Index in Testicular Diffuse Large B-Cell Lymphoma

Pauli Vähämurto, Marjukka Pollari, Michael R. Clausen, Francesco d'Amore, Sirpa Leppä and Susanna Mannisto

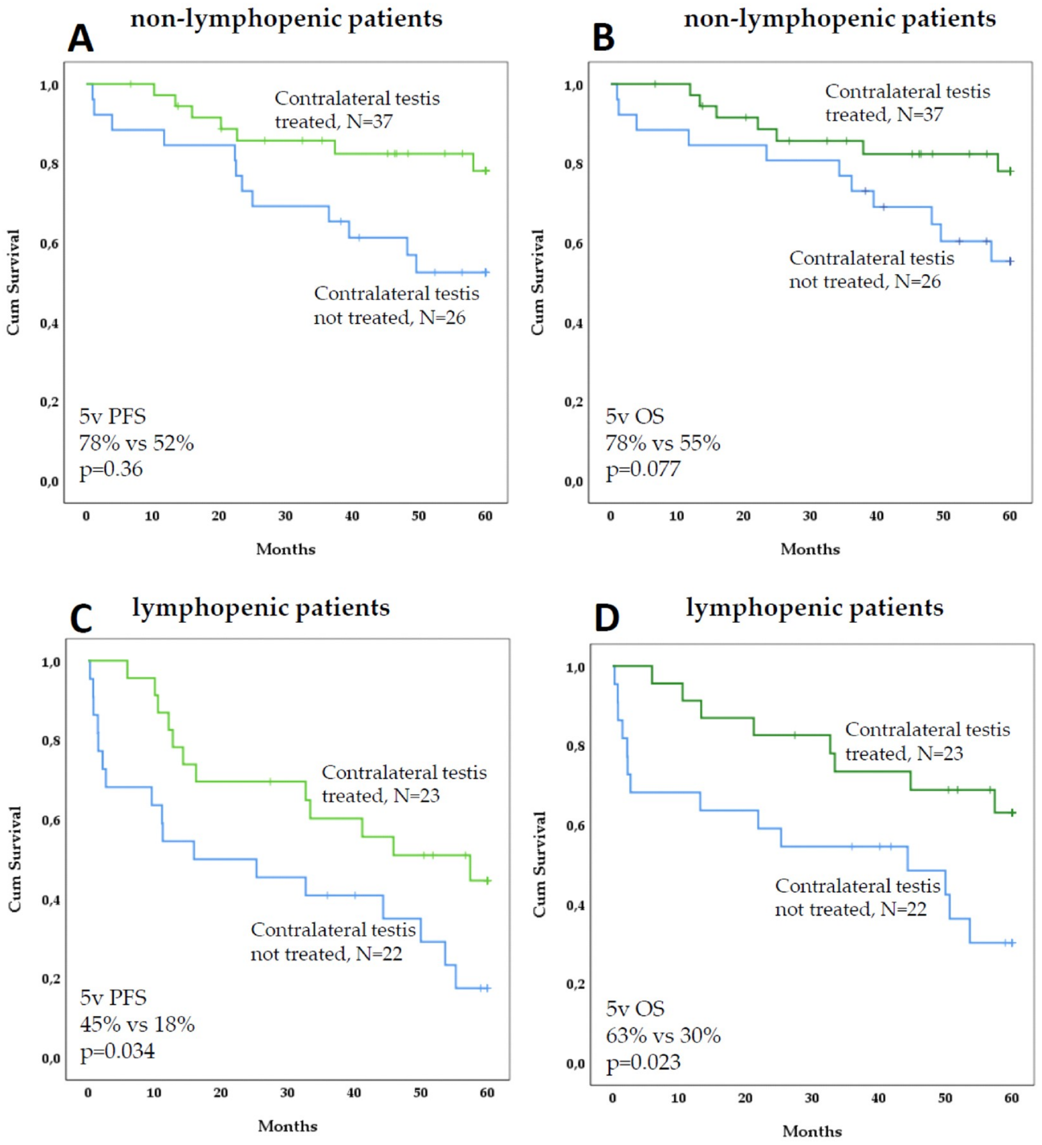

Figure S1. Kaplan-Meier estimates of the impact of treatment of the contralateral testis on survival among non-lymphopenicrituximab treated patients (**A, B**); lymphopenicrituximab treated patients (**C, D**). Association to improved progression free survival (PFS) and overall survival (OS) is seen only among non-lymphopenicpatients (**A, B**).

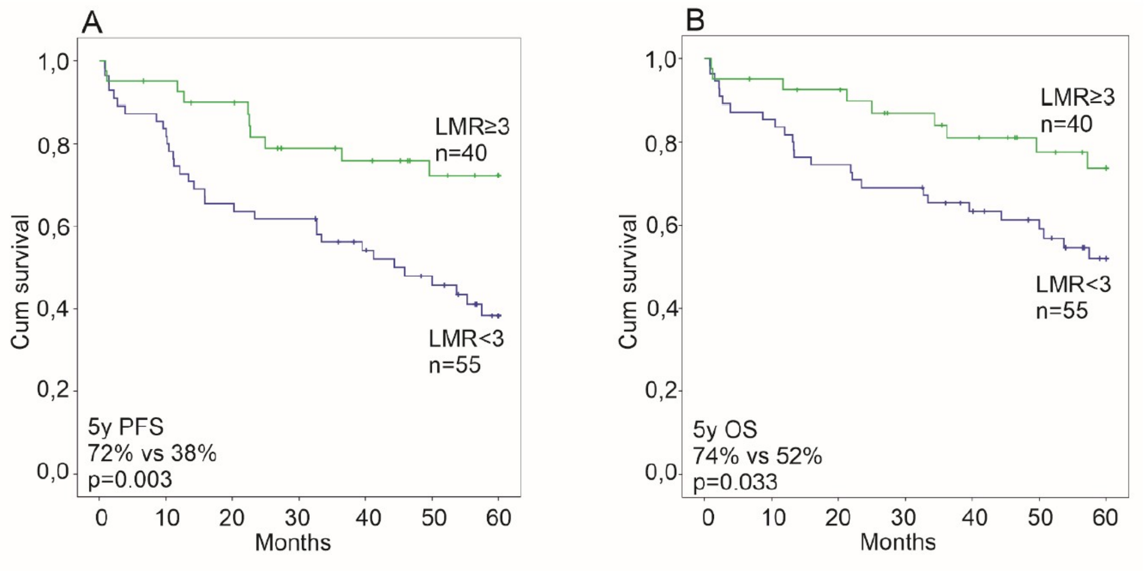

Figure S2. Kaplan-Meier estimates of the impact of lymphocyte to monocyte ratio (LMR) among rituximab treated patients showing improved overall survival (OS) (**A**) and progression free survival (PFS) (**B**) among patients with LMR ≥ 3:1.

Table S1. Cox regression analyses on multivariate level of rituximab treated patients showing independent association of lymphocyte-monocyte ratio and other baseline characteristics and treatment parameters with outcome.

	PFS, Hazard Ratio (95% CI)	*p*	OS, Hazard Ratio (95% CI)	*p*
low LMR	3.500 (1.630–7.515)	**0.001**	3.406 (1.463–7.928)	**0.004**
Age >60	1.439 (0.530–3.911)	0.475	3.454 (0.780–15.302)	0.103
Stage ≥3	1.349 (0.568–3.208)	0.498	1.486 (0.599–3.685)	0.393
ECOG ≥2	2.504 (1.074–5.937)	**0.034**	2.312 (0.914–5.845)	0.077
LDH high	1.348 (0.582–3.123)	0.486	1.298 (0.513–3.287)	0.582
extranodal sites >1	2.768 (1.170–6.549)	**0.020**	3.833 (1.574–9.331)	**0.003**
iv CNS dir	0.304 (0.134–0.693)	**0.005**	0.197 (0.075–0.516)	**0.001**
Treatment of the contralateral testis	0.274 (0.135–0.553)	**<0.001**	0.239 (0.104–0.552)	**0.001**

PFS, progression free survival; OS, overall survival; LMR, lymphocyte-to-monocyte ratio; Age >60 y, patients over 60 years; ECOG, Eastern Cooperative Oncology Group performance score; LDH, lactate dehydrogenase level; IPI, International Prognostic Index; iv CNS dir, intravenous central nervous system directed treatment. Statistically significant p-values are bolded.

Table S2. Cox regression analyses on multivariate level of rituximab treated patients showing independent association of high lactate dehydrogenase to lymphocyte ratio and other baseline characteristics and treatment parameters with outcome.

	**PFS, Hazard Ratio (95% CI)**	***p***	**OS, Hazard Ratio (95% CI)**	***p***
High LDL/ALC	1.957 (0.927–4.130)	0.078	1.368 (0.598–3.125)	0.458
Age >60	1.558 (0.598–4.056)	0.364	3.866 (0.911–16.404)	0.067
Stage 1–2	0.921 (0.444–1.912)	0.826	0.981 (0.452–2.131)	0.961
ECOG 0–1	1.687 (0.804–3.542)	0.167	1.808 (0.824–3.964)	0.140
extranodal sites >1	2.966 (1.406–6.257)	**0.004**	3.622 (1.645–7.976)	**0.001**
iv CNS dir	0.427 (0.213–0.857)	**0.017**	0.308 (0.137–0.693)	**0.004**
Treatment of the contralateral testis	0.491 (0.271–0.889)	**0.019**	0.495 (0.255–0.964)	**0.039**

LDH/ALC, lactate dehydrogenase to absolute lymphocyte count ratio; PFS, progression free survival; OS, overall survival; Age >60 y, patients over 60 years; ECOG, Eastern Cooperative Oncology Group performance score; IPI, International Prognostic Index; iv CNS dir, intravenous central nervous system directed treatment. Statistically significant p-values are bolded.

© 2020 by the authors. Licensee MDPI, Basel, Switzerland. This article is an open access article distributed under the terms and conditions of the Creative Commons Attribution (CC BY) license (http://creativecommons.org/licenses/by/4.0/).
